# Supplementary material for: Protection of Galacto-Oligosaccharide against E. coli O157 Colonization through Enhancing Gut Barrier Function and Modulating Gut Microbiota
Source: Foods. 2020 Nov 21;9(11):1710. doi: 10.3390/foods9111710 (PMC7700679; doi:10.3390/foods9111710)
Supplement: Supplementary file 1 [file foods-09-01710-s001.pdf]

foods

MDPI

Supplementary File:

Table S1. Primer Sequences for qPCR

Gene	Forward Primer（5′→3′）	Reverse Primer（5′→3′）
Stx1	GAAGAGTCCGTGGGATTACG	AGCGATGCAGCTATTATTAA
β-actin	GGCTGTATTCCCCTCCATCG	CCAGTTGGTAACAATGCCATGT
IL-17	GCTCCAGAAGGCCCTCAGA	AGCTTTCCCTCCGCATTGA
IL-6	ACTTCCATCCAGTTGCCTTCTTG	AGCTGGATGCTCTCATCAGG
IL-1β	TTGACGGACCCCAAAAGAT	AGCTGGATGCTCTCATCAGG
TNF-α	GCTCTGTGAAGGGAATGGGTGTT	GCTCTGTGAAGGGAATGGGTGTT
MUC2	CTGTGCCAATGGCCTCAAAC	GCCCATCGAAGGTGACAAAG
Occludin	GGTCTCTACGTGGATCAATATTTGTA	AACCCCAGGACAATGGCTA
ZO1	CTCATCTCCAGTCCCTTACC	CTCCTCCAGGCTGACATTAG
Claudin	TCGGCCAACACCATCATCAG	TACAACCCAGCTCCCATCTC

qPCR condition: The PCR was performed as follows: initial melting temperature at 95 °C for 60 s, 40 cycles of denaturation at 95 °C for 15 s, and 60 °C for 40 s, draw melting curve at the end.

Table S2. Concentration of SCFAs in Feces of Each Group.

Group	Acetic Acid (μmol/g)	Propionic Acid (μmol/g)	Butyric Acid (μmol/g)	Valeric Acid (μmol/g)	Total SCFAs (μmol/g)
CK	43.14 ± 8.98	12.84 ± 2.80	14.13 ± 2.64	5.09 ± 1.06	75.21 ± 9.33
MC	43.09 ± 6.47	12.89 ± 2.88	13.91 ± 3.21	5.16 ± 1.17	75.07 ± 6.16
GOS	51.70 ± 12.62	13.81 ± 3.70	16.24 ± 4.97	5.25 ± 2.24	87.00 ± 16.61

Data were expressed as mean ± SD (n = 8). There were no significant differences between the groups (p > 0.05).
